# The Mediating Role of the Gut Microbiota in the Physical Growth of Children

**DOI:** 10.3390/life12020152

**Published:** 2022-01-20

**Authors:** Magdalena Durda-Masny, Joanna Ciomborowska-Basheer, Izabela Makałowska, Anita Szwed

**Affiliations:** Institute of Human Biology and Evolution, Faculty of Biology, Adam Mickiewicz University, 61-614 Poznan, Poland; joanna.ciomborowska@amu.edu.pl (J.C.-B.); izabela.makalowska@amu.edu.pl (I.M.)

**Keywords:** gut microbiota, growth, infants, children

## Abstract

Gut microbiota succession overlaps with intensive growth in infancy and early childhood. The multitude of functions performed by intestinal microbes, including participation in metabolic, hormonal, and immune pathways, makes the gut bacterial community an important player in cross-talk between intestinal processes and growth. Long-term disturbances in the colonization pattern may affect the growth trajectory, resulting in stunting or wasting. In this review, we summarize the evidence on the mediating role of gut microbiota in the mechanisms controlling the growth of children.

## 1. Introduction

The gut microbiota is a group of microorganisms, mainly bacteria, yeast, fungi, bacteriophages, and other viruses [1], as well as protozoa and archaea [2,3], which form a complex ecosystem in the human gastrointestinal (GI) tract. The intestinal microbiota performs several functions, including participating in the activation and maturation of the immune system, regulating the immune response, maintaining intestinal epithelial homeostasis, providing protection against the invasion of opportunistic pathogens, synthesizing enzymes necessary for the breakdown of complex plant polysaccharides, and taking part in hormonal regulation [4,5,6,7]. Dysbiosis, reduced diversity in the gut microbial community, or microbial immaturity are involved in several intestinal and metabolic diseases that affect growth [8]. It is essential that an intense increases in body size, especially in infancy, occur in parallel with the dynamic succession of gut microbiota [9]. In recent years, more attention has been paid to the study of the mediating role of gut microbiota in shaping the process of physical growth in children. In this paper, we review the mechanisms through which the gut microbiota affects the process of growth in children.

## 2. Physical Growth of Children

The physical growth of children is defined as an irreversible and constant increase in body size (length or height and weight) and in the size of organs [10]. It is a dynamic, complicated, and long process that continues throughout infancy, childhood, and adolescence [11]. Tracking the growth trajectories of children is important, as they provide essential indicators of infant and childhood development and can predict potential adult health outcomes [12]. A vast body of evidence suggests that physical growth in the earliest stages of ontogenesis not only sets the pattern for adult size but also establishes a biological scaffold for adult health through the profound effects that nutrition, illness, ecology, and social environment have on early development [13]. Undoubtedly, the group of factors affecting children’s development in the first years of life also includes the set of microorganisms that colonize the intestines at that time. Based on the standards developed by the World Health Organization [14], there are three types of growth trajectory in children: standard or normal growth, delayed growth, and rapid growth. The assessment of growth is performed based on anthropometric measurements of the child and a comparison of the obtained values to growth standards. The standards depict normal human growth under optimal environmental conditions and can be used to assess children regardless of ethnicity, socioeconomic status, and type of feeding [14]. Research suggests that children with normal growth trajectories are more likely to experience better health outcomes than those with abnormal growth [15,16]. Abnormalities in child growth refer to deviations in height, weight, or head circumference. Delayed growth is recognized when these deviations are below the standards [17]. Stunting refers to a child with a height-for-age Z-score (HAZ) two standard deviations (−2 SD) below the standard. Wasting, on the other hand, refers to a child with a weight-for-height Z-score (WHZ) < −2 SD below the standard. More specifically, moderate acute malnutrition (MAM) is recognized when the WHZ of the child is between −2 and −3 SD below the standard, whereas severe acute malnutrition (SAM) is recognized when the child’s WAZ ranks below −3 SD from the median of the WHO reference growth standards [18]. Both SAM and MAM usually develop between 3 and 24 months of life [19], which is the period in which the pattern of the gut microbiota is established. Rapid growth is defined when the deviations are above the standards [14,17]. Other very useful tools for assessing the nutritional status of children are the cut-off points developed by Cole et al. [20,21], who defined the ranges of underweight, norm, overweight, and obesity.

**Figure 1:**
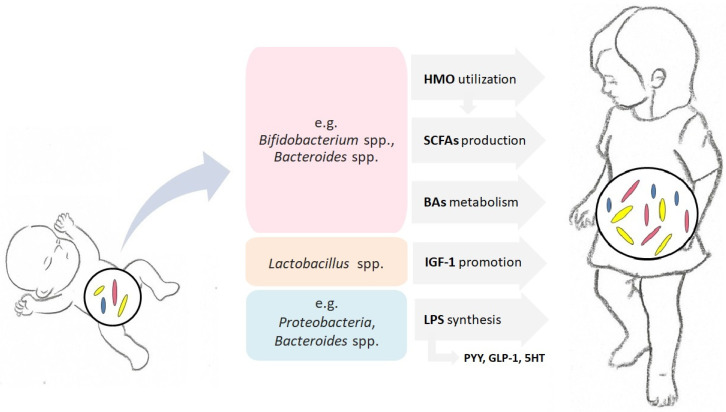
Selected mechanisms through which gut microbes affect the process of growth in children. The mediating role of intestinal microbiota in child growth consists of participation in metabolic processes through the production of biologically active metabolites, mainly short-chain fatty acids (SCFAs), in the metabolism of human milk oligosaccharides (HMO) and bile acids (Bas), participation in the hormonal activity of the host through indirect impact on the hormone levels, including insulin-like growth factor-1 (IGF-1), peptide YY (PYY), glucagon-like peptide-1 (GLP-1), or serotonin (5HT), and stimulation of the innate immune system, as well as the initiation of the inflammatory process (through the synthesis of lipopolysaccharides, LPS).

The consequences of abnormal growth rates are varied. Evidence suggests that rapid weight gain in early life is associated with several determinants of metabolic syndrome (e.g., cardiovascular disease and type 2 diabetes) in early adulthood [22,23]. The subsequent consequences of delayed growth include poor cognitive and psychosocial outcomes [24]. The best documented causes of impaired linear growth in children include lowered or increased body mass index, unfavorable prenatal conditions, and infections [25]. All the aforementioned factors affecting the course of growth in children have been well explored. In recent years, it has become clear that a complex of intestinal microbes is also involved in the process of shaping the growth trajectories of children. The intestinal microbiota takes part in a wide range of mechanisms regulating the process of growth, which is reflected in the rate and magnitude of changes in child body size over time (Figure 1).

## 3. Gut Microbiota in the Early Stages of Human Growth

There are presumptions that the process of microbiota colonization begins in utero, most likely through the translocation of bacteria or their antigens to the fetus from the maternal gastrointestinal tract, then from the mother and the environment at delivery, and that it continues throughout the first three years of life [26,27,28]. The development and maturation of the intestinal microbiota are influenced by many factors, such as placental inflammation, maternal infection during pregnancy [29], course of pregnancy, length of pregnancy, type of delivery [30], perinatal condition, hospital environment and length of hospitalization [31], feeding method, use of antibiotics, [32], lifestyle, and geographical factors [33]. From birth to the age of three years, the gut microbiota gradually develops into the adult microbiota [34]. After the third year of life, the intestinal microbiota reaches maturity. Therefore, the fetal period and the first three years of life are considered the critical window for the formation of the microbial colonization pattern. Any disturbances in the process of colonization of the gut microbiota may have a long-term effect on host growth, development, and health later in life [35,36,37]. This is also a key period for infant growth, with significant gains in body length and weight as well as head and chest circumference [37,38]. Both prenatal development and development in early childhood are characterized by the rapid maturation of metabolic, endocrine, nervous, and immune pathways that strongly influence and support the growth and development of the child. These pathways run in parallel and are highly interdependent, and their disturbance by adverse environmental factors, such as infection, may distort the trajectory of a child’s growth and development [37]. As demonstrated by Stewart et al. [39], there are three distinct phases of microbiome succession: a developmental phase (months 3–14), a transitional phase (months 15–30), and a stable phase (months 31–46). Recent studies have shown that the predominant early colonizers of the healthy infant’s gut are maternal fecal bacteria, mainly *Bifidobacterium* and *Bacteroides*, and *Clostridium* during the following six months [26,40,41,42,43]. Studies have also demonstrated that *Bacteroides* are associated with increased gut diversity and faster intestine maturation [39]. Natural childbirth has been shown to be significantly related to microorganisms reflecting the mother’s vaginal flora, such as *Bacteroides*, *Lactobacillus*, and *Prevotella* [30,39]. Vaginal microbiota with low diversity and rich in *Lactobacillus* has been connected to birth at term and appropriate birth weight [37]. In turn, in children born by cesarean section, bacteria inhabiting mainly the skin surface, *Staphylococcus, Corynebacterium*, and *Propionibacterium*, predominate [30]. Cesarean section is one of the most significant disrupting factors of the proper colonization process [44]. Another important factor associated with infant gut microbiota colonization is breast milk [42]. Approximately 25–30% of the infant’s gut microbiota originates from the mother’s milk [45]. Breastfeeding is related to the predominance of *Bifidobacterium* and *Lactobacillus* [46], and the cessation of breastfeeding results in faster gut microbiome maturation, marked by *Firmicutes* [39]. When modified milk is used, the dominance of *Bacteroides* and *Clostridium* is noticeable [46].

## 4. Microbiota Succession and Growth of Children

The results of the studies carried out thus far allow us to conclude that the course of colonization of the intestinal microbiota in the early stages of ontogenesis has far-reaching consequences for the linear and ponderal growth of children. The most extensive research exploring the aforementioned relationship was performed in the context of malnutrition. Smith et al. [47] conducted an elegant long-term study on the role of the gut microbiota in Malawian twin pairs discordant for kwashiorkor, a form of SAM. Both children in these pairs of twins were treated with a ready-to-use therapeutic food (RUTF) based on peanuts, which resulted in a transient maturation of metabolic function in the kwashiorkor microbiome. In the next stage of the research, fecal microbiota were transplanted from several discordant pairs into gnotobiotic mice. It was noticed that mice that received transplants from children with kwashiorkor and were fed the Malawian diet experienced significant weight loss and disturbances in carbohydrate and amino acid metabolism. The administration of RUTF only transiently ameliorated the observed effects. This finding led to the conclusion that the intestinal microbiome is a casual factor in this type of severe malnutrition, which was also confirmed by Pham et al. [48].

In the study by Subramanian et al. [49], the process of intestinal microbial succession in a cohort of children living in the urban slum of Dhaka, Bangladesh, who exhibited consistently healthy growth during the first two years of life, was analyzed. With the use of a machine-learning-based approach to 16S rRNA datasets, the authors assessed the most age-discriminatory taxa, including *Bifidobacterium longum* and *Streptococcus thermophilus*. In the next step, they developed the index of gut microbiota maturity, the so-called microbiota-for-age Z-score (MAZ). In the same study, the MAZ among children with severe acute malnutrition (SAM) (WHZ < −3) was also analyzed. The results revealed that compared to the healthy group, children with SAM had a significantly lower MAZ. This indicates that the gut microbiota of the children with SAM was significantly less mature than that of the healthy children [49].

The results of a longitudinal birth cohort study conducted by Dinh et al. [50] on a small group of children in the urban slum community in Vellore, India showed that a reduced relative abundance of *Bifidobacterium longum* and *Lactobacillus mucosae*, in addition to an elevated relative abundance of *Desulfovibrio* ssp., was associated with stunting. It has been shown that stunted children possess a different gut microbiota composition to non-stunted children. The intestinal microbial ecosystem of stunted children was enriched in inflammogenic taxa, whereas that of the non-stunted group was enriched in probiotic bacterial species. The results of other studies conducted in the suburbs of Chandigarh, India also indicated that the pattern of gut microbiota profile in malnourished children differs from that of healthy subjects [51]. It has been documented that the intestinal microbiota of healthy children is characterized by a significantly higher abundance of *Bifidobacterium* in comparison to children suffering from SAM. Interesting results were also obtained by Gough et al. [52], who conducted research on a group of children from Malawi and Bangladesh. It has been shown that reduced microbiota diversity is associated with stunting severity and that overgrowth of *Acidaminococcus*, as well as elevated glutamate-fermenting microbes, may contribute to future growth deficits in already malnourished children. In contrast to the aforementioned studies, Méndez-Salazar et al. [53] investigated differences in the composition of the gut microbiota in both undernourished and obese children from Mexico. The undernourished children demonstrated a significantly higher abundance of *Firmicutes* and *Lachnospiraceae* than the obese children, while the *Proteobacteria* was overrepresented in the obese subjects. Therefore, it can be concluded that the course of intestinal microbiota succession plays an important role in the regulation of early-life growth.

## 5. Microbiota and Host Metabolism Regulation

Several studies have shown that the early-life microbiota plays a mediating role in host metabolic processes. Gut microbes are essential intermediaries in a wide range of mechanisms, including energy harvest, fat storage, regulation of lipid and glucose metabolism, induction of low-grade inflammation, gut barrier function, control of satiety through gut hormones, and interactions with host genetics [54,55]. To put it simply, the contribution of microbiota to the aforementioned mechanisms is based both on its metabolites, mainly (but not only) short-chain fatty acids (SCFAs), and on the activity of lipopolysaccharides (LPS) and bile acids [55].

### 5.1. Microbiota and Short-Chain Fatty Acids

Some taxa of enteric microbiota possess the ability to ferment nondigestible carbohydrates. The major products of this fermentation are short-chain fatty acids, primarily acetate, propionate, and butyrate, which play various roles not only in the gastrointestinal tract but also distantly [56,57]. It has been documented that SCFAs can regulate host cell activity via two mechanisms: by binding to G-protein-coupled receptors (GPCRs) or by acting as inhibitors of histone deacetylase [58]. Receptors and transporters for SCFAs are expressed in a vast range of cells from the gastrointestinal tract to the immune and nervous systems [59,60]. The effects of GPCR activation differ greatly depending on the cell in which they are expressed [61]. Butyrate serves as a major energy source for enterocytes [62] and acts as a histone deacetylase inhibitor [63], contributing to the maintenance of barrier integrity and reducing the levels of intestinal inflammation markers [64,65,66]. Butyrate, acetate, and propionate activate GPCRs and thus trigger a wide range of intracellular transduction cascades, including those that modulate hormonal activity [67]. It has been documented that concentrations of SCFAs in the cecum are higher in conventionally raised mice than in germ-free (GF) animals [64], whereas the colonization of GF mice results in increases in SCFA concentrations [68]. At the same time, the use of broad-spectrum antibiotics results in a decrease in SCFA concentrations in conventionally raised mice [68]. This proves that SCFA concentrations are associated with the presence of gut microbes.

### 5.2. Microbiota and Lipopolysaccharides

The most significant events in the development of host immunity occur in the earliest stages of development [69]. The intense changes in the composition of the microbial community that take place in the infant gut make this period a “window of opportunity” [42]. For this reason, any abnormalities in intestinal succession may have far-reaching consequences for the functioning of the immune system, manifested by increased sensitivity to infections or inflammatory diseases [70,71], which may impair growth. The maturation of innate immunity is characterized by the colonization of gram-negative bacteria, mainly *Proteobacteria* and *Bacteroides* spp. The common feature of these two taxa is the presence of lipopolysaccharides (LPS) in the outer cell membrane [72], a pathogen-associated molecular pattern (PAMP) that can stimulate both the innate immune system and nonimmune cells and initiate the inflammatory process [73,74,75]. LPS are potent ligands for Toll-like receptors (TLRs), mainly Toll-like receptor 4 (TLR4), which are expressed in many cells of the body [76], including epithelial enteroendocrine (*EE*) cells. LPS can activate TLRs in EEs and thus stimulate the secretion of metabolically active hormones, such as peptide YY (PYY) [77], glucagon-like peptide-1 (GLP-1) [78], or serotonin (5HT) [79]. PYY inhibits intestinal motility and increases the absorption rate of nutrients through the intestinal epithelium [80]. GLP-1 potentiates glucose-stimulated insulin secretion in β-cells [81], whereas 5HT participates in the regulation of intestinal motility and fluid secretion [79]. Elevated concentrations of LPS in the blood trigger a condition termed “metabolic endotoxemia” [82,83]. Numerous metabolic disorders, including insulin resistance, type II diabetes, and obesity, are possibly related to this state [84]. Elevated LPS concentrations may result from increased intestinal permeability associated with compositional disturbances in gut microbiota [83]. Endotoxemia can, therefore, contribute to low-grade inflammation, disturbed mucosal barrier integrity, and impaired glycemic control through perturbations in gut hormone secretion, which characterize metabolic syndrome [76,84]. It has been documented that obese children present higher levels of *Proteobacteria* and that there is a positive correlation between *Proteobacteria*, *Bacteroides*, and fat intake [53]. Therefore, it can be concluded that the excessive ponderal growth that characterizes obese children may be associated with an increased abundance of bacteria producing LPS. However, it is not entirely clear what role different LPS-producing bacteria play in the pathophysiology of excessive weight gain or early education of immunity in children [55].

### 5.3. Microbiota and Bile Acids

Bile acids (BAs) constitute the primary components of bile and are secreted into the small intestine in conjugated forms. The main function of BAs is to aid the solubilization, and thus absorption, of dietary lipids [76]. Most bile acids are reabsorbed in the ileum and returned to the liver, whereas unabsorbed BAs enter the large intestine and are deconjugated and metabolized into secondary BAs by gut microbes [85] via bile salt hydrolase (BSH), which is widely present among intestinal microbiota [86]. Moreover, BAs act as ligands for a nuclear farnesoid X receptor (FXR) as well as for G-protein-coupled bile acid receptor-1 (TGR5). The activation of these receptors initiates a variety of signaling cascades relevant to the regulation of lipid, sugar, and energy metabolism [87]. Tanaka et al. [88] conducted a longitudinal study on a group of Japanese children in which they monitored the succession of the gut bacterial community and its association with the fecal BA profile in the first three years of life. Evidence from this research provided insights that neonates undergo a massive transition in their BA profiles during lactation, which overlaps with the transition of the gut microbiota profiles. Moreover, it has been documented that perturbations of BAs or microbiota profiles (or both) during the first three years of life may result in negative metabolic consequences. However, the associations between bile acids and microbiota succession in infants need to be further explored.

## 6. Microbiota and Human Milk Oligosaccharides

In early infancy, the diversity of microbiota is low, especially in breastfed babies. During this period, the composition of the gut microbial ecosystem is dominated by species whose role is to participate in the metabolism of human milk oligosaccharide (HMO) [45]. HMOs are a unique-to-humans, structurally complex, and diverse group of glycans, which are present in human milk in high quantities [89]. They are key bioactive components of mothers’ milk [90] and provide numerous health-promoting effects by inhibiting pathogen attachment to epithelial cells (ECs) [91], promoting the growth of specific microbes, modulating intestinal immune responses and patterns of gene expression in intestinal epithelial cells [92]. HMOs can indirectly increase SCFA production, and these elevated levels are mediated by bifidobacterial species [93]. HMOs are composed of five monosaccharides: glucose (Glc), galactose (Gal), N-acetylglucosamine (GlcNAc), fucose (Fuc), and sialic acid N-acetyl-neuraminic acid (Neu5Ac). As HMO compositions can vary, breastfed infants are exposed to structurally different HMOs [94]. The degradation of HMOs to low-molecular-weight oligosaccharides depends on the enzymes produced by bacteria. Next, low-molecular-weight oligosaccharides can be reduced to monosaccharides via carbohydrate-degrading enzymes and converted to SCFAs [95].

The presence of bacteria capable of HMO enzymatic degradation, which the host organism cannot perform on its own, is, therefore, crucial in the production of SCFAs or other organic compounds that may be beneficial to the host during infancy. Moreover, the HMOs present in mothers’ milk stimulate the growth of bacteria that possess the ability to break down these compounds (the so-called bifidus factor). Breastfed infants exhibit significantly higher levels of *Bifidobacterium* and *Lactobacillus* and lower levels of pathogenic bacteria than formula-fed infants [96]. Additionally, breastfed infants and formula-fed infants differ in their SCFA concentrations. Specifically, breastfed infants are characterized by higher levels of acetate and lactate and lower levels of propionate and low or absent levels of butyrate [94]. Until recently, HMOs were not present in the formulas administered to children who, for some reason, could not be breastfed, which resulted in a decrease in the percentage of *Bifidobacterium* in the developing community of gut microbes (due to the lack of a factor stimulating their development).

An excellent study by Charbonneau et al. [97] shed light on the mechanisms through which HMOs interact with gut microbes to regulate growth. The content of breast milk HMOs from mothers in two Malawian birth cohorts was characterized. One cohort included infants who exhibited healthy growth, whereas the other cohort included infants who were severely stunted. The mothers of the stunted infants exhibited a significantly lower abundance of HMOs in breast milk at six months. The reduced HMO content mainly concerned sialylated HMOs, including sialyllacto-N-tetraose b, which was revealed to be the most growth-discriminatory [97]. Next, GF mice and piglets were colonized with a consortium of cultured bacterial strains from the stool of a severely stunted infant. The animals were fed a representative Malawian diet. One group of animals received supplementation of sialylated bovine milk oligosaccharides (S-BMO) that was structurally similar to sialylated HMOs, whereas the other group did not. Supplementation of the animals with S-BMO stimulated weight gain and bone volume. This effect was not evident in the GF animals. The results established that sialylated HMOs trigger a microbiota-dependent promotion of growth.

HMO composition depends on maternal genetics. Mothers who are carriers of an active fucosyltransferase 2 (*FUT2*) gene, categorized as secretors, produce more HMOs [94]. The composition of the infant gut community depends on the maternal secretor status; in the children of secretors, *Bifidobacterium* is more abundant [98]. However, the fact that the mother is a carrier of *FUT2* per se is not associated with infant growth [99]. The relationship between HMO composition and the growth of children has already been demonstrated in many studies [89,100,101]

## 7. Microbiota and Growth Hormone/Insulin-like Growth Factor-1 Axis

The major controller of growth in children is the activity of the somatotropic axis. When a child experiences a nutritional deficiency over a long period of time, growth hormone (GH) resistance develops, and the child becomes stunted. The liver and peripheral tissues, including muscles, produce insulin-like growth factor-1 (IGF-1), which promotes systemic and organ growth. IGF-1 is a key mediator of skeletal growth, acting in an endocrine, paracrine, and autocrine manner [102]. The production of IGF-1 is controlled by GH, secreted by the anterior pituitary gland. One of the most interesting mechanisms that could explain the relationship between gut microbiota composition and linear and ponderal growth of children involves the role of intestinal microbes in the promotion of growth hormones. The evidence for this concept comes from the research conducted by Shin et al. [103] and Storelli et al. [104] on *Drosophila melanogaster*. In the first study, the colonization of germ-free (GF) larvae with *Acetobacter pomorum* was shown to restore the developmental rate and body size of the host [103]. The results of the second study showed that the strain of *Lactobacillus plantarum* was sufficient to stimulate the growth of the host under nutrient-poor conditions [104]. Schwarzer et al. [105] documented the impact of gut microbiota on the growth of mice in the neonatal period. The studies compared the growth parameters of wild-type (WT) and germ-free (GF) infant male mice fed a standard breeding diet through early adulthood. The GF mice exhibited significantly lower rates of growth and weight gain than the WT animals. Bone mass and femur length were also lower in the GF mice. Furthermore, the GF mice presented reduced IGF-1 and insulin-like growth factor binding protein-3 (IGFBP-3) levels and reduced expression of *Igf1* and *Igfbp3* in the liver compared to the WT animals. When the mice were fed a nutrient-depleted diet, the GF animals exhibited greater weight loss and a significant decrease in the expression of *Igf1* and *Ghr* genes in the liver and muscles compared to the WT mice. This evidence indicates that the GF mice developed GH resistance. Additionally, as a result of injections of recombinant IGF-1 (rIGH-1) in the GF mice, there was an increase in body weight and body length, whereas in the WT mice, this effect was not observed. This led to the conclusion that the gut microbiota stimulates growth by facilitating IGF-1 production and activity. In addition, the colonization of GF *Drosophila* [104,106,107] and GF mice [68,105] with conventional microbiota, including a specific strain of *L. plantarum,* restored normal growth and production, as well as IGF-1 activity. Moreover, in GF animals, in which GH resistance was observed, it also restored the sensitivity of peripheral tissues to this hormone. A similar effect of growth stimulation in the presence of *Lactobacillus rhamnosus* was also observed in zebrafish [108].

It is not fully understood how the microbiota stimulates IGF-1 production. It seems, however, that the link between the gut microbiota and the regulation of IGF-1 of the host are byproducts produced by these microbes, whose effects on host IGF-1 levels have been observed [102]. This link has become apparent since *Lactobacillus* emerged as one of the key microbes affecting IGF-1 levels, and the members of this genus are well-known SCFA producers [109]. In the study of Yan et al. [68], supplementation of SCFAs from antibiotic-treated mice increased the IGF-1 level. Therefore, SCFAs, which are metabolized by gut bacteria from otherwise indigestible-fiber-rich diets, seem to be the most important link between intestinal microbes and the host IGF-1 level, but other mechanisms through which gut microbes affect bone growth probably exist [68,103].

### Microbiota and Other Hormones

In addition to its mediating role in regulating the activity of the somatotropic axis, the gut microbiota indirectly influences the levels of other hormones in the host, such as PYY, GLP-1, leptin, ghrelin, and serotonin, and it also plays a mediating role in the activity of the adrenal axis [109,110]. Therefore, it is not an exaggeration to conclude that the gut microbiota can be considered an endocrine organ.

## 8. Conclusions

The intestinal microbiota is involved in a wide range of processes that regulate the growth of children. The mediating role of gut microbes in growth consists of participation in metabolic processes through the secretion of enzymes and the production of biologically active metabolites (e.g., SCFAs) as well as in the metabolism of HMO and BAs, participation in the hormonal activity of the host through indirect impact on the hormone levels (including IGF-1, PYY or GLP-1), and participation in immune responses and processes leading to inflammation (LPSs). All the aforementioned mechanisms do not exhaust the complexity of the relationship between the course of intestinal succession and the course of children’s growth. There is no doubt, however, that due to the wide range of interactions between the gut microbiota and growth processes, disturbances in gut microbial succession may negatively affect patterns of child growth.

## Data Availability

Not applicable.
